# Hypofractionated irradiation of infra-supraclavicular lymph nodes after axillary dissection in patients with breast cancer post-conservative surgery: impact on late toxicity

**DOI:** 10.1186/s13014-015-0480-y

**Published:** 2015-08-20

**Authors:** Marina Guenzi, Gladys Blandino, Maria Giuseppina Vidili, Deborah Aloi, Elena Configliacco, Elisa Verzanini, Elena Tornari, Francesca Cavagnetto, Renzo Corvò

**Affiliations:** Department of Radiotherapy – IRCCS A.O.U. San Martino – IST, Genoa, Italy; Department of Medical Physics – IRCCS A.O.U. San Martino – IST, Genoa, Italy; Department of Oncological Rehabilitation – IRCCS A.O.U. San Martino – IST, Genoa, Italy

**Keywords:** Breast cancer, Hypofractionation, Regional Nodal Irradiation, Toxicity

## Abstract

**Background:**

The aim of the present work was to analyse the impact of mild hypofractionated radiotherapy (RT) of infra-supraclavicular lymph nodes after axillary dissection on late toxicity.

**Methods:**

From 2007 to 2012, 100 females affected by breast cancer (pT1- T4, pN1-3, pMx) were treated with conservative surgery, Axillary Node Dissection (AND) and loco-regional radiotherapy (whole breast plus infra-supraclavicular fossa). Axillary lymph nodes metastases were confirmed in all women. The median age at diagnosis was 60 years (range 34–83). Tumors were classified according to molecular characteristics: luminal-A 59 pts (59 %), luminal-B 24 pts (24 %), basal-like 10 pts (10 %), Her-2 like 7 pts (7 %). 82 pts (82 %) received hormonal therapy, 9 pts (9 %) neo-adjuvant chemotherapy, 81pts (81 %) adjuvant chemotherapy. All patients received a mild hypofractionated RT: 46 Gy in 20 fractions 4 times a week to whole breast and infra-supraclavicular fossa plus an additional weekly dose of 1,2 Gy to the lumpectomy area. The disease control and treatment related toxicity were analysed in follow-up visits. The extent of lymphedema was analysed by experts in Oncological Rehabilitation.

**Results:**

Within a median follow-up of 50 months (range 19–82), 6 (6 %) pts died, 1 pt (1 %) had local progression disease, 2 pts (2 %) developed distant metastasis and 1 subject (1 %) presented both. In all patients the acute toxicity was mainly represented by erythema and patchy moist desquamation. At the end of radiotherapy 27 pts (27 %) presented lymphedema, but only 10 cases (10 %) seemed to be correlated to radiotherapy. None of the patients showed a severe damage to the brachial plexus, and the described cases of paresthesias could not definitely be attributed to RT. We did not observe symptomatic pneumonitis.

**Conclusions:**

Irradiation of infra-supraclavicular nodes with a mild hypofractionated schedule can be a safe and effective treatment without evidence of a significant increase of lymphedema appearance radiotherapy related.

## Background

In high-risk node-positive women with breast cancer, national and international guidelines (AIRO/NCCN/DEGRO) recommended to extend radiotherapy to lymph nodal area (apex axilla - III level and/or supraclavicular regions) after axillary dissection to improve loco-regional control and survival, especially in the presence of additional clinical and biological risk factors [1–4]. The standard given dose post conservative surgery remains 50 Gy in 25 fractions over 5 weeks and it is recommended a boost to the tumor bed to further improve the local control [5]. Several randomized trials proved that in selected low-risk patients, shorter treatment regimens (3 to 4 weeks) with a hypofractionated schedule may be safe and effective with comparable medical outcome and cosmesis [6, 7]. A task force group endorsed by the American Society for Radiation Oncology (ASTRO) asserted that data are now sufficient to support the use of hypofractionated whole breast irradiation (HF-WBI) for selected patients with early-stage breast cancer [8] and recently the same association has recommended the administration of such therapeutic schedule [9]. Published data supportive of hypo-fractionated schedules are limited to breast irradiation and only few clinical trials are available on regional lymph node irradiation with short schemes [10]. The hypo-fractionated loco-regional radiotherapy scheme used in our Institute is not particularly compressed in time but provides a smaller number of fractions (20 versus 25), during which the boost to the tumor bed is simultaneously administered once-a-week, reducing the patient accesses [11]. This is a retrospective study about one hundred consecutive patients treated with mild hypofractionated locoregional radiotherapy. The aim of the present work was to analyse the impact on late toxicity of this hypofractionated scheme in order to assess safety and efficacy in infrasupraclavicular area.

## Methods

From 2007 to 2012, one hundred patients affected by breast cancer were treated with conservative surgery, axillary node dissection (AND) and loco-regional (whole breast plus infra-supraclavicular fossa) hypofractionated RT. The median age at diagnosis was 60 years (range 34–83). Histology was ductal carcinoma in 76 patients (76 %). Characteristics of patients are shown in Table 1. Tumors were classified according to molecular characteristic as shown in Table 2. Axillary lymph nodes metastasis were confirmed in all women (81 patients post conservative surgery and 9 patients at diagnosis, before neo-adjuvant chemotherapy). Patients received systemic therapy: hormonal in 82 patients (82 %), neo-adjuvant chemotherapy in 9 patients (9 %), adjuvant chemotherapy in 81 patients (81 %). All the administered chemotherapy schedules contained anthracycline derivatives and taxanes. In agreement with several randomized phase III trials which have shown to improve local control with mild side effects and acceptable cosmetic outcome [5] in our center all the patients receive a boost dose. The selected group of patients examined in this analysis received hypofractionated RT with concomitant boost as described in detail in a previous published experience [11], Our treatment schedule was 46 Gy in 20 fractions 4 times a week to whole breast and infra-supraclavicular fossa (from Monday to Friday with a day off on Wednesday) with concomitant additional weekly dose of 1,2 Gy (preferibly delivered on Monday) to the lumpectomy area (total boost dose of 6 Gy in 5 fractions once a week). As reported by our previous paper, using the Linear-Quadratic cell survival model we assumed that 46Gy in 2.3 Gy fractions is equivalent to 50Gy in 2.0 Gy fractions as shown in Table 3 [11]. The four fractions per week schedule is commonly used in our Department in order to optimize the clinical and dosimetric activities, by also allowing an easier integration of palliative treatments [11–13]. Most treatments (81 %) were delivered by linear accelerator Varian Clinac 2100CD (RX, 6–15 MV), and the remaining 19 % by Tomotherapy Hi-Art® System (Accuray®, RX 6MV) when it was necessary to improve coverage of the target or minimize dose to normal tissue in selected patients in which a steep dose gradient was required. A planning computed tomography (CT GE Lightspeed Ultra) scan was made for each patient, positioned on a wing-board with both arms raised above the head. Patients were scanned from the level of the larynx to the level of the upper abdomen, including both lungs, with a scan thickness and index of 5 mm [11]. Four tattoos were made on the thoracic skin to allow the repositioning of the patient during treatment sessions. The whole breast clinical target volume (WB-CTV) included the glandular breast tissue of the ipsilateral breast. The WB-CTV did not extend into the pectoralis major, nor the ribs, and did not include the skin [11]. The three-dimensional tissue volumes containing the supraclavicular (SCV) infraclavicular (IFV) and III level lymph nodes were defined on CT scans by using readily identifiable anatomic landmarks on the guide of two published papers (Table 4) [14]. In patients treated by linear accelerator (81 %), whole breast radiation was delivered by opposed tangential beams and infra-supraclavicular fossa by two or more opposing fields (main beam energy: 6 MV and, when it is necessary to improve target coverage, 15 MV). The gantry angles, the use of multileaf collimator, wedges and additional subfields were employed to achieve optimal dose distribution and maximal organ at risk avoidance (heart, left anterior descending coronary artery, ipsilateral lung, esophagus, thyroid and spinal cord). Constraints for the organs at risk are shown in Table 5, overdosages were limited within 5 %, this means that global Dmax is mantained lower than 105 % and this is possible using sub fields with MLC closed on overdosage regions. Tomotherapy Hi-Art® treatments were delivered using a 0.403 pitch, Modulator Factor (MF) ranging between 1.5–4, field dimension 2.5 cm. It was impossible to exclude the brachial plexus from the radiation fields due to its proximity to the lymph nodes region. Treatment Planning was performed in order to give 95 % of the prescribed dose to PTV; in three-dimensional (3D) conformal Radiotherapy an Eclipse v.7.3.10 Varian Treatment Planning System was used while Helical tomotherapy planning was performed with the HI-Art® Tomotherapy inverse planning system (Madison, WI, USA). Portal films were taken at least once during the first treatment day and compared to digitally reconstructed radiographs (DRR) and simulator images to ensure accurate set up. In Tomotherapy a pre-treatment daily image guidance with megavoltage (MV) CT scan was performed. After matching with the kilovoltage planning CT, corrections for translations and rotation around longitudinal axis (roll) were done. The disease control and treatment-related toxicity were analysed in follow-up visits, performed at 6, 12 months after therapy and, subsequently, annually. Particular attention was paid to late side effects correlated to radiation of infra-supraclavicular fossa. The extent of lymphedema was analysed at the department of Oncological Rehabilitation of our Institute, by measuring upper limbs circumference at two different locations obtained 10 cm above and below the antecubital fossa in both upper extremities and comparing the values with those of the contralateral arm, as suggested by other authors [15]. Mild, moderate and severe arm lymphedema were defined as a difference of 0.5–2 cm, 2.1–3 cm and >3 cm respectively, in the circumference at one or more measurement sites between the treated and untreated sides [15]. Patients were followed by Oncological Rehabilitation Unit to appearance of lymphedema according to its severity.

**Table 1:** Patients’ characteristics

Characteristic	N	%
Age		
<50	24	24 %
50-59	23	23 %
>60	53	53 %
Histology		
DCI	75	75 %
LCI	15	15 %
Others	10	10 %
Pathological tumor stage		
pTis	2	2 %
pT1	51	51 %
pT2	38	38 %
pT3	2	2 %
pT4	2	2 %
Not evaluable	5	5 %
Pathological nodal stage		
pN0	9	9 %
pN1	25	25 %
pN2	43	43 %
pN3	23	23 %
Grading		
G1	4	4 %
G2	54	54 %
G3	38	38 %
Not evaluable	4	4 %
Ki67		
≤14 %	27	27 %
>14 %	71	71 %
Not evaluated	2	2 %

*DCI* ductal carcinoma invasive, *LCI* lobular carcinoma invasive

**Table 2:** Molecular characteristics of patients

Molecular subtype	N	%
Luminal A	59	59 %
Luminal B	24	24 %
C-erb +++	7	7 %
Basal like	10	10 %

**Table 3:** BED comparison between standard and explored RT schedule [11]

RT schedule	BED tumor control α/β _4_		BED acute effects α/β _10_		BED fibrosis α/β _1.7_		BED vascular damage α/β _2.5_	
W.B. = whole breast	W.B.	B.S	W.B.	B.S	W.B.	B.S	W.B.	B.S
B.S. = tumor bed side								
60 Gy/30 F/6 W	75	90	60	72	109	131	90	108
(50 Gy + 10 Gy seq.boost)								
52 Gy/20/F/5 W (46 Gy + 6 Gy cc.boost)	72	87	57	66	108	135	88	108
UK START TRIAL A	75	75	55	55	120	120	95	95
41.6 Gy/13 F/5 W								
UK START TRIAL A	68	68	51	51	108	108	86	86
39 Gy/13 F/5 W								

*boost* concomitant boost, *seq.boost* sequential boost, *F* fractions, *W* weeks

**Table 4:** Regional Nodal Contours: anatomical boundaries [14]

	Anteriorly	Posteriorly	Superiorly	Inferiorly	Medially	Laterally
SCV	bounded by the deep surface of the sternocleidomastoid and the deep cervical fascia	Lat: the anterior and medial borders of the anterior scalene muscle.		the posterior border was defined by the subclavian artery	lateral edge of the trachea excluding the thyroid gland and thyroid cartilage superiorly	
		Med: medially to the carotid artery and internal jugular vein				
IFV	the deep surface of the pectoralis major muscle	subclavian-axillary artery	the most superior aspect of the pectoralis minor muscle	level of the insertion of the clavicle into the manubrium	lateral edge of the clavicle	medial border of the pectoralis minor muscle

*SCV* supraclavicular fossa, *IFV* infraclavicular fossa

**Table 5:** Constraints for organs at risk in adjuvant radiotherapy of early breast cancer

Organ at risk	Normofractionation 2 Gy per fraction\5fr\week	Hypofractionated schedule 2,3 Gy per fraction\4 fr\week
LADCA	V_20Gy_ = 0 %	V_19Gy_ = 0 %
Heart	V_20Gy_ = 10 % V40Gy =5 %	V_19Gy_ = 0 %
Ipsilateral Lung	V_20Gy_ = 25 % (exclusive periclavicular LN)	V_19Gy_ = 25 % (exclusive periclavicular LN)
	V_20Gy_ = 35 % (inclusive periclavicular LN)	V_19Gy_ = 35 % (inclusive periclavicular LN)
Spinal cord	Max 45 Gy	Max 42 Gy

*LADCA* left anterior descending coronary artery, *LN* lymph nodes

## Results

Within a median follow-up of 50 months (range 19–82), 6 patients (6 %) died, one patient (1 %) had local progression disease, 2 patients (2 %) developed distant metastasis and 1 (1 %) subject presented both. In all patients the acute toxicity, according to the RTOG/EORTC classification [16], was mainly represented by erythema and patchy moist desquamation. In 27 patients (27 %) a lymphedema was recorded during the visit at the Department of Oncological Rehabilitation of our institute: 15 cases (15 %) as a likely consequence of chemotherapy and surgery because they appeared before radiotherapy. In only three cases, a preexisting lymphedema was worsened by radiotherapy. In 12 patients (12 %) it appeared after the end of radiotherapy: in 2 patients as a result of disease progression to the lymph nodes, in the remaining 10 patients appears to be related to radiotherapy. The time of onset of lymphedema in the latter 10 cases was very variable (from 1 to 48 months from the end of radiotherapy). The cases of radio induced lymphedema were classified as follows: 4 mild, 2 moderate and 4 severe. These patients were treated with manual lymph drainage and compression therapy. Post-RT, three patients showed paresthesias of upper limbs, two bilaterally, already present before starting radiation treatment, the last one ipsilateral, which resolved spontaneously in few months. All patients received chemotherapy regimens containing taxanes, of which paresthesias are one of the side effects. Treatment was generally well tolerated: a limited number of severe lymphedema (4 %), none of the patients showed a severe damage to the brachial plexus, the described cases of paresthesias could not definitely be attributed to RT and we did not observed symptomatic pneumonitis.

## Discussion

The aim of the present work is to investigate the outcomes of a mild HF-WBI for the treatment of breast cancer when delivered to women who need irradiation to the infra-supraclavicular lymph nodal region. Our results seem to suggest that this hypofractionated schedule to the breast and the regional nodes is as effective as a standard RT regimen in the absence a significant increase of side effects. This evidence is of note since many concerns emerge when an altered fractionated regimen is proposed for the irradiation of locoregional node areas. As reported in the literature, the irradiation of the lymph node area may increase the risk of side effects such as radiation pneumonitis, lymphedema and brachial plexopathy. Since acute and late side effects are correlated to the volume of irradiated parenchyma, regional nodal radiotherapy can increase the pneumonitis rates (1.3 % vs 0.2 %) [17]. In order to reduce the risk of radio-induced pneumonitis, internationally approved protocols should be strictly applied [18]. Lymphedema may represent a standard complication after any axillary surgery. Its incidence can be difficult to define, as no standardized definition of lymphedema exists. The onset of lymphedema is related different causes including the extension of axillary dissection (5–15 % after dissection, 1–3 % after sentinel node biopsy), obesity, the association with chemotherapy and RT delivery [19–23]. Shah et al., provides the incidence of lymphedema according to the extent of RT after dissection, reporting a rate from 2–35 % after breast irradiation and an increased incidence to 9–65 % in the case of loco-regional irradiation [20]. The different findings of lymphedema may be related not only to individual clinical situations and radiation techniques applied (extension of treatment volumes, type of planning, others) but also to the different methods of evaluation and classification used. Inter-limb circumferential discrepancy has been the most widely used outcome measure. Moreover, brachial plexopathy is a major concern with loco-regional RT: the induced damage may be related to dose per fraction, total dose, and volume irradiated. Using standard fractionation schedule (50Gy/25fr) the rates of brachial plexopathy are less than 5 % [10]. In recent years several schemes of hypofractionated WBI were developed and tested in randomized trials. The results with long-term follow-up demonstrated that the adoption of a shorter schedule may be safe and effective with comparable medical outcome and cosmesis to the standard irradiation: no statically significant differences both in local control and aesthetic result emerged in women who received the standard or the hypofractionated breast radiation [6, 7].

However, few data are still available as regards the late effects of hypofractionated radiotherapy when extended to the regional lymph nodes. A recent update of START A and START B evaluated the loco-regional RT in a limited group of patients and neither the 5 week nor the 3 week treatment developed significantly worse normal tissue impacts: the assessments of arm and shoulder effects showed no evidence of a detrimental effect for the hypofractionated schedules [7]. In 2011 Yarnolds et al. already showed no radio-induced brachial plexus toxicity after hypofractionated irradiation of the axilla and/or supraclavicular fossa. Those Authors stated that the START B regimen (40 Gy in 15 fractions/3 weeks) is equivalent to 47 Gy in 2.0-Gy fractions if the α/β value for brachial plexus is 2.0 Gy or to 49 Gy in 2.0-Gy fractions, if α/β = 1.0 Gy [24]. Haffty and Buchholz commented on the absence of side effects in the small group of patients (116pts of 2215, 7 %) enrolled in the START B trial and receiving regional hypofractionated RT: they confirmed that these results are consistent with modelling of normal tissue effects, which predicts that 40 Gy in 15 fractions should be as safe as the standard scheme for all normal tissues [25]. The results of several randomized studies demonstrated the feasibility and effectiveness of the hypofractionated WBI. Although limited, other data collected during hypofractionated regional nodes irradiation did not reveal increased toxicity as compared to standard fractionation RNI [10]. Badiyan N. et al. reviewed prospective and randomized data to analyse the efficacy and toxicity of hypofractionated radiation schedules in breast cancer with RNI to the axilla and supraclavicular regions. They noted that RNI with standard fractionation is associated with increased toxicity compared to WBI alone but current data do not support an increased rate of toxicity with hypofractionated RNI compared with standard fractionation RNI [10]. The outcomes of our investigation seem to confirm what emerges from the literature: patients treated with hypofractionated RT (46Gy in 20 fractions with 4 times a week) to the whole breast and infra-supraclavicular fossa (plus once weekly concomitant boost dose of 1,2 Gy to the lumpectomy area) did not show a higher rate of side effects, in 10 patients the lymphedema was radio induced, which only 4 severe. We observed no symptomatic pneumonitis, no radiation-related damage to the brachial plexus and only 11 % of lymphedema. The favorable results obtained with this fractionation can be the basis for investigating new radiation schemes with a smaller number of fractions administered in reduced times.

## Conclusions

Mild hypofractionated WBI delivered in 20 fractions represents a safe and effective treatment for the infrasupraclavicular node areas. ASTRO board reported that shorter treatment schedules can significantly benefit patients in terms of convenience, acceptance of therapy, and cost. They suggest to do not initiate whole-breast radiation therapy as a part of breast conservation therapy in women age ≥50 with early-stage invasive breast cancer without considering shorter treatment schedule [9]. The limited experience available on hypofractionated scheme extended to lymph nodes do not seem to show an increased toxicity. In our department the adoption of this mild hypofractionated loco-regional radiation schedule in the context of a policy of gradual reduction of the numbers of the fractions has shown to be safe when delivered on locoregional nodal areas. This preliminary evidence should be further investigated and confirmed by large-scale prospective studies.
